# Association of smoking with knee osteoarthritis structural defects and symptoms: an individual participant data meta-analysis

**DOI:** 10.1038/s41598-024-80345-x

**Published:** 2024-11-22

**Authors:** Zubeyir Salis, Amanda Sainsbury

**Affiliations:** 1 Division of Rheumatology, Geneva University Hospital and Faculty of Medicine, University of Geneva, HUG Av. de Beau-Séjour 26, 1206 Geneva, Switzerland; 2 The University of Western Australia, School of Human Sciences, Perth, WA Australia

**Keywords:** Knee osteoarthritis, Smoking, Ageing, Osteoarthritis, Risk factors

## Abstract

Prior meta-analyses have suggested a protective link between smoking and knee osteoarthritis (KOA), but they relied on aggregate data, potentially obscuring the true relationship. To address this limitation, we conducted an Individual Participant Data (IPD) meta-analysis using data from three large cohorts: the Osteoarthritis Initiative (OAI), the Multicenter Osteoarthritis Study (MOST), and the Cohort Hip and Cohort Knee (CHECK) study. Participants from 16 centers in the USA and Netherlands were categorized as current, former, or never smokers. We assessed six outcomes, three related to structural changes over 4–5 years of follow-up, and three related to changes in KOA symptoms over 2–2.5 years, 5 years, and 7–8 years of follow-up. First, the incidence of radiographic KOA was evaluated in 10,072 knees, defined as having a Kellgren-Lawrence grade ≥ 2 (‘radiographic KOA’) at follow-up but not at baseline. Second, the progression of radiographic KOA was evaluated in 5274 knees, defined as an increase in Kellgren–Lawrence grade between baseline and follow-up in knees that had radiographic KOA at baseline. Third, the incidence of symptomatic KOA was evaluated in 12,910 knees, defined as having radiographic KOA in addition to symptoms at follow-up but not at baseline. Fourth, fifth, and sixth, we investigated changes between baseline and all follow-ups in scores for the Western Ontario and McMaster Universities Osteoarthritis Index (WOMAC) subscales of pain, disability, and stiffness (in 2640 knees). There were no differences between smoking groups in any of these six outcomes. Our study, leveraging data from three large cohorts and the advantages of IPD, finds no evidence that smoking offers any protection against KOA, refuting the notion that smoking may benefit joint health.

## Introduction

Smoking is a significant health risk and impacts nearly every organ in the body. It contributes to many diseases and disabilities^1^. Interestingly, smoking has been reported to have potentially favorable effects on certain conditions, including ulcerative colitis^2^, Parkinson’s disease^3^, and knee osteoarthritis (KOA)^4^. Given that KOA is a primary cause of chronic pain and disability among older adults^5,6^, understanding the factors contributing to its development and progression is crucial to devising effective prevention strategies.

Observational studies on the relationship between smoking and KOA have yielded mixed results^7–30^, with several suggesting that smoking may be protective against the development and progression of structural defects seen in radiography, such as joint space narrowing, cartilage loss, and bone spur growth^7–19^, as well as being protective against progression to total knee replacement^20^. However, these conclusions are subject to debate due to variations in statistical methods, exposure measures, and outcome measures across studies. This variability is expected to be addressed in meta-analyses, and previous meta-analyses^31–34^ have suggested that smoking might indeed exert protective effects on the development and progression of KOA. However, these meta-analyses^31–34^ used the method of aggregate data meta-analysis^35^, where information from each underlying study is summarized. Given the potential differences in statistical methods, exposure measures, and outcome measures among the summarized studies, aggregate data meta-analysis could be obscuring the true association between smoking and KOA^36–38^, affecting the accuracy of the conclusions.

To address the above-mentioned limitations of aggregate data meta-analysis, a more robust approach is proposed: individual participant data (IPD) meta-analysis^37^. In contrast to aggregate data meta-analysis where summary statistics from each study are combined and analyzed, IPD meta-analysis involves combining and analyzing the original raw data from each participant across all included studies. This allows for consistent application of statistical methods, exposure definitions, and outcome measures across the entire dataset. By standardizing these elements, IPD meta-analysis minimizes the risk of heterogeneity and enhances the precision of the estimates. This approach also enables more sophisticated analyses, such as adjusting for covariates at the individual level and conducting subgroup analyses, which are often limited in aggregate data meta-analysis. IPD meta-analyses have proven pivotal in shaping clinical guidelines by providing more robust and reliable evidence^39^. For example, they have led to significant changes in the United Kingdom (UK) National Institute for Health and Care Excellence (NICE) guidelines on antibiotic prescriptions for ear infections and chemoradiation treatments for cervical cancer, where previous recommendations based on aggregate data meta-analyses were overturned^40,41^.

Despite the demonstrated advantages of IPD meta-analysis over aggregate data meta-analysis, no study to our knowledge has used this method to investigate the relationship between smoking and KOA. Our study thus aims to address this gap. Contrary to previous findings, we hypothesize that smoking does not exert protective effects on KOA.

## Methods

### Data sources

We obtained publicly available data from three cohorts: the Osteoarthritis Initiative (OAI)^42^; the Multicenter Osteoarthritis Study (MOST)^43^; and the Cohort Hip and Cohort Knee (CHECK) study^44^. The OAI, conducted at four clinical centers in the United States of America (USA) between February 2004 and May 2006, enrolled 4,796 adults (with 9592 knees) aged 45–79 years who were “with or at risk of KOA”. The MOST, conducted at two USA centers between April 2003 and April 2005, registered 3026 adults (with 6052 knees) aged 50–79 years who were “with or at risk of developing KOA”. CHECK, conducted at ten Dutch centers from October 2002 to September 2005, enrolled 1002 adults (with 2004 knees) aged 45–65 years who were “with persistent knee or hip complaints suggestive of early osteoarthritis”. The informed consent documents and protocols of these cohorts were approved by the corresponding local institutional ethics review boards. The current study was not registered upfront in a registry.

### Study outcomes

We investigated the association between smoking and structural defects of KOA as well as symptoms of KOA.

Structural defects of KOA were assessed using Kellgren–Lawrence (KL) grades^45^. In clinical practice, the KL grading system is commonly used to assess KOA severity by providing an aggregate score of structural defects observed by radiography. These defects include joint space narrowing, osteophyte formation, subchondral sclerosis, and bone deformity^46^. KL grades range from 0 to 4, with higher KL grades indicating worse structural defects in the knee joint. A knee with a KL grade ≥ 2 was considered in this study to have “radiographic KOA”.

Symptoms of KOA were assessed using the Western Ontario and McMaster Universities Osteoarthritis Index (‘WOMAC’)^47^. The WOMAC is a widely used, validated instrument that is divided into three subscales: pain, disability, and stiffness. The WOMAC pain score ranges from 0 to 20, the WOMAC disability score ranges from 0 to 68, and the WOMAC stiffness score ranges from 0 to 8. Higher scores on each subscale indicate greater severity of KOA symptoms. We considered a knee with a sum of the WOMAC pain and disability subscale scores of ≥ 12 to have moderate to severe symptoms of KOA^48^. The WOMAC stiffness subscale score was not included in this definition because, although stiffness is an important symptom of KOA, it has low reliability in clinical assessments^49^.

We assessed six outcomes encompassing the structural defects and symptoms of KOA. Of these six outcomes, three included measures of structural defects (and were binary outcomes), and three focussed only on symptoms (and were continuous outcomes).

Our three outcomes involving measures of structural defects (binary outcomes) were: (1) the incidence of radiographic KOA, defined as a KL grade ≥ 2 at follow-up, in knees that had no such evidence at baseline; (2) the progression of radiographic KOA, defined as an increase in KL grade from baseline to follow-up in knees already classified as having radiographic KOA at baseline; and (3) the incidence of symptomatic KOA, defined as the presence of both radiographic KOA and moderate to severe symptoms of KOA (i.e., sum of the WOMAC subscales for pain and disability ≥ 12 as defined above) at follow-up in knees that did not meet these criteria at baseline. These outcomes were investigated at follow-up at 4-to-5 years from all three cohorts (OAI, MOST, and CHECK). For the OAI, follow-up was at 4 years, because only a limited number of radiographs were available beyond 4 years. For MOST and CHECK, follow-up was at 5 years, because they did not have any radiographs at 4 years.

Our three outcomes that focussed only on symptoms (continuous outcomes) were: changes in symptoms of KOA as assessed by the WOMAC subscale scores for (1) pain, (2) disability, and (3) stiffness between baseline and follow-up. Each of these three changes from baseline in symptoms was evaluated at 2 years, 5 years, and 8 years for the OAI and CHECK cohorts. For MOST, the changes were evaluated at 2.5 years, 5 years, and 7 years. There were no data beyond 7 years in MOST. Thus, the follow-ups investigated were 2-to-2.5 years, 5 years, and 7-to-8 years.

### Inclusion and exclusion criteria for the selection of knees used for analyses in this study

**Fig. 1:**
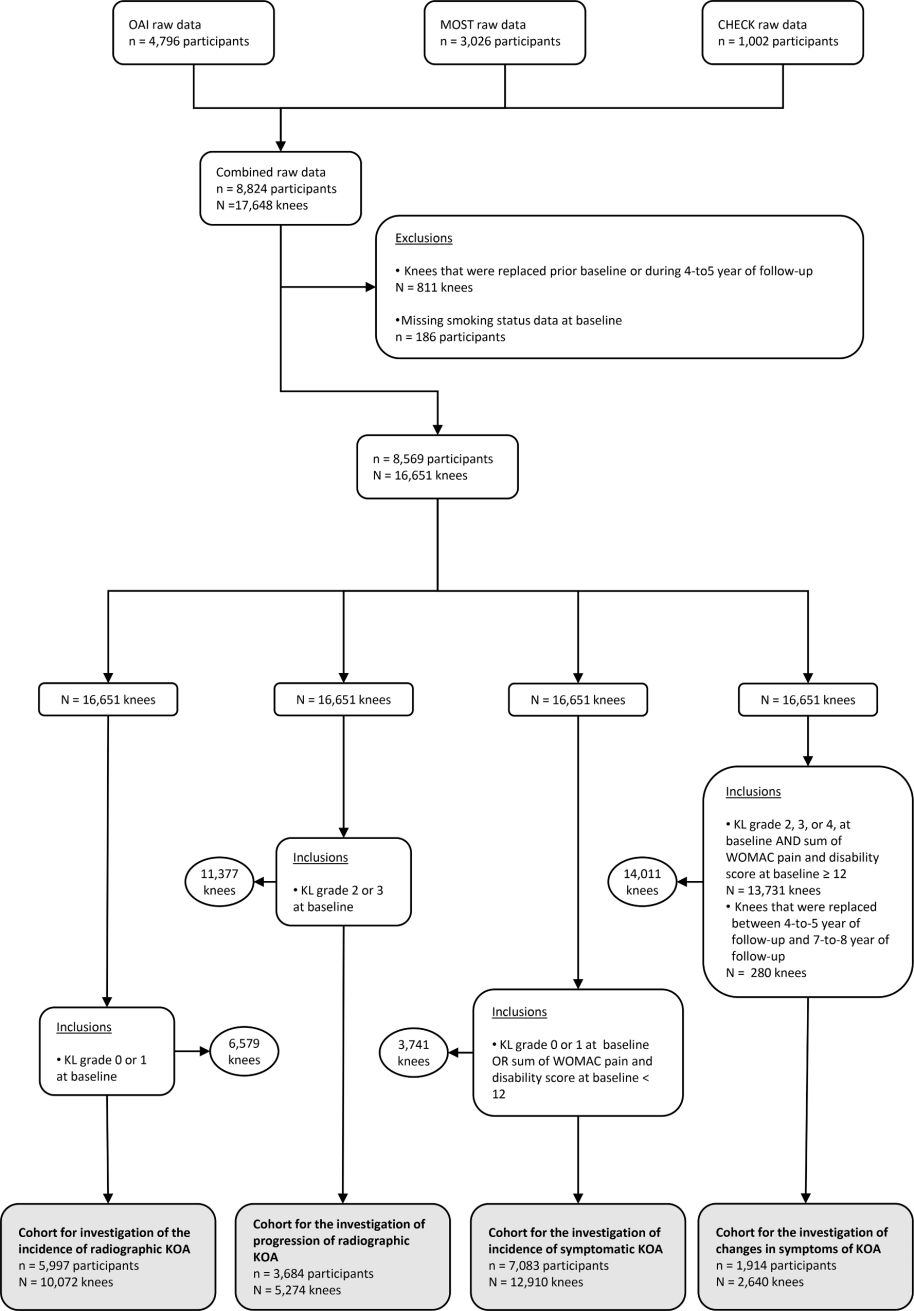
Selection of knees for our investigations of the symptoms and structural defects of knee osteoarthritis. *CHECK* Cohort for Hip and Cohort for Knee, *KL* Kellgren–Lawrence, *KOA* Knee Osteoarthritis, *MOST* Multicohort Osteoarthritis Study, *OAI* Osteoarthritis Initiative, *WOMAC* Western Ontario and McMaster Universities Arthritis Index.

From the OAI, MOST, and CHECK cohorts (17,648 knees from 8824 participants in total), we created four cohorts of knees for investigation of our six outcomes: (1) the “cohort for investigation of the incidence of radiographic KOA”; (2) the “cohort for investigation of the progression of radiographic KOA”; (3) the “cohort for investigation of the incidence of symptomatic KOA”; and (4) the “cohort for investigation of the changes in symptoms of KOA” (Fig. 1). First, we excluded knees that had been replaced prior to baseline and during the first 4-to-5 years of follow-up (811 knees) (Fig. 1). We further excluded participants who had missing smoking status data at baseline (186 participants) (Fig. 1). The remaining 16,651 knees from 8569 participants (Fig. 1) were then sorted into the four cohorts. Firstly, the cohort for investigation of the incidence of radiographic KOA included knees with a KL grade of 0 or 1 at baseline, totalling 10,072 knees from 5997 participants (Fig. 1). Secondly, the cohort for investigation of the progression of radiographic KOA included knees with radiographic KOA at baseline (i.e., a KL grade of 2 or 3 at baseline), totalling 5274 knees from 3684 participants. Although radiographic KOA was defined as a KL grade ≥ 2 (i.e., a KL grade of 2, 3, or 4), we excluded knees with a KL grade of 4 at baseline from this cohort, as further progression in their KL grade is not possible (Fig. 1). Thirdly, the cohort for investigation of the incidence of symptomatic KOA included all knees that did not meet the criteria for symptomatic KOA at baseline. Thus, this cohort was composed of (1) knees without radiographic KOA (i.e., a KL grade of 0 or 1), regardless of their WOMAC scores, or (2) knees with radiographic KOA (i.e., a KL grade ≥ 2) but with no to low symptoms of KOA (i.e., a sum of the WOMAC subscales for pain and disability < 12). This cohort for investigation of the incidence of symptomatic KOA comprised 12,910 knees from 7083 participants (Fig. 1). Fourthly, the cohort for investigation of changes in symptoms of KOA included knees that met the criteria for symptomatic KOA at baseline (i.e., a KL grade ≥ 2 and a sum of the WOMAC subscales for pain and disability ≥ 12). As we had already excluded the knees that had been replaced between baseline and the first 4-to-5 years of follow-up, for this cohort, we further excluded the knees that had been replaced between 4-to-5 years of follow-up and 7-to-8 years of follow-up. In total, the cohort for investigation of the changes in symptoms of KOA comprised 2,640 knees from 1914 participants (Fig. 1).

### Exposure

Our exposure of interest was participants’ self-reported smoking status at baseline, categorized as “current smoker”, “former smoker”, or “never smoker”. For CHECK, we reclassified the responses from participants who reported that they “smoke every day or occasionally” at baseline as “current smoker”, and who reported that they “used to smoke every day or occasionally” at baseline as “former smoker”.

### Statistical analyses

In our analyses, we utilized the one-stage IPD meta-analysis approach^50^. In this approach, all the participant‐level data from all underlying studies are analyzed in a single step. We conducted data quality checks to help ensure reliability of IPD from the OAI, MOST, and CHECK cohorts. The key baseline variables (i.e., age, sex, education status, employment status, KOA severity, and smoking status) were harmonized to ensure consistent definitions across datasets. We conducted data completeness checks to screen for potential outliers and logical inconsistencies (e.g., implausible values for age, KL grades, or WOMAC scores). No outliers or logical inconsistencies were identified in the data.

We investigated the association between smoking status and the three binary outcomes (i.e., incidence of radiographic KOA, progression of radiographic KOA, and incidence of symptomatic KOA, all over the 4-to-5 years of follow-up) using generalized estimating equations for the binomial family with a logistic link function (i.e., logistic regression)^51^. We considered the left and right knee of each participant as clustered data and assumed an exchangeable correlation structure within participants (i.e., the correlations between measurements on both knees were treated as equal). The estimates were adjusted for sex, race, study cohort, and baseline values of age, education status (above high school degree or not), employment status (working in a paid job or not), and KL grade. Additionally, for the outcome of the incidence of symptomatic KOA, we adjusted the estimate for the sum of the WOMAC subscale scores of pain and disability at baseline, as well as for the use of analgesics. While we adjusted our estimates for baseline KL grade to account for initial differences in disease severity between groups, we also conducted additional analyses without this adjustment, to evaluate whether this adjustment influenced our findings.

In our primary analyses, we excluded knees that had been replaced either prior to baseline or during follow-up, as mentioned earlier in the Methods section. However, since knee replacement can indicate KOA incidence or progression, we repeated these analyses of binary outcomes, treating knee replacement as an occurrence of the binary outcome under investigation (i.e., incidence of radiographic KOA, progression of radiographic KOA, and incidence of symptomatic KOA). Furthermore, we conducted an additional analysis to examine the number of participants where both knees, only one knee, or neither knee showed any of the three binary outcomes. This analysis was conducted in an attempt to gain further insight into any potential effect of smoking status on KOA, under the hypothesis that if smoking is having an effect on KOA, then it would be more likely to affect both knees in a single person than to affect only one of the two knees per person.

For analysis of our three continuous outcomes (i.e., changes in symptoms of KOA), we used mixed linear effect modeling. We explored the influence of quadratic and cubic transformations of time (i.e., follow-up year) in the models to account for potential non-linear changes over time. As these transformations were statistically significant, we retained the quadratic and cubic forms of time in the models, in addition to the linear form of time. To address the inherent correlation between measurements in the left and right knee within each participant, we specified a random effect for participants. This effectively clustered the data for left and right knees within the same individual, acknowledging their interdependence, similar to what we did for the analyses of binary outcomes, where we considered the left and right knee of each participant as clustered data and assumed an exchangeable correlation structure within participants. Moreover, we incorporated a random effect of the knee side to handle the dependence in repeated observations on the same knee over the follow-up duration and a random slope for the effect of time to allow for differences in trajectory over time. Smoking status at baseline, time, and the interaction between smoking status at baseline and time (in linear, quadratic and cubic forms) were entered as predictor variables in the models. These interactions were retained as they were statistically significant. The estimates were additionally adjusted for sex, race, study cohort, and baseline values of age, education status, employment status, and use of analgesics. We chose not to adjust our estimates for baseline WOMAC scores to avoid the risk of inducing a false statistical association between smoking status and the change in WOMAC scores^52,53^, as current and former smokers had higher WOMAC scores than non-smokers in our study (Tables 1 and 2). To investigate whether adjusting our estimates as described above had any effect on the results, we conducted additional analyses by adjusting for baseline WOMAC scores (in addition to adjusting for the covariates specified above).

**Table 1:** Baseline characteristics of knees in the cohort for investigation of the incidence of radiographic KOA and cohort for investigation of the progression of radiographic KOA, stratified by smoking status of participants.

Baseline characteristics	Cohort for investigation of the incidence of radiographic KOA			Cohort for investigation of the progression of radiographic KOA		
	Current smoker	Former smoker	Never smoker	Current smoker	Former smoker	Never smoker
Participants	n = 480 (8.00)	n = 2477 (41.30)	n = 3040 (50.69)	n = 242 (6.57)	n = 1462 (39.69)	n = 1980 (53.75)
Knees	N = 829 (8.23)	N = 4170 (41.40)	N = 5073 (50.37)	N = 351 (6.66)	N = 2086 (39.55)	N = 2837 (53.79)
Age, years	55.67 ± 6.81	60.62 ± 8.37	59.42 ± 8.43	58.10 ± 7.94	63.72 ± 8.25	62.52 ± 8.81
BMI, kg/m^2^	27.27 ± 4.77	28.08 ± 4.77	28.00 ± 4.85	29.91 ± 4.48	30.44 ± 5.40	30.50 ± 5.72
Race						
White	644 (77.68)	3703 (88.80)	4404 (86.81)	215 (61.25)	1664 (79.77)	2184 (76.98)
Other	185 (22.32)	467 (11.20)	669 (13.19)	136 (38.75)	422 (20.23)	653 (23.02)
Sex						
Male	363 (43.79)	1737 (41.65)	1839 (36.25)	141 (40.17)	894 (42.86)	940 (33.13)
Female	466 (56.21)	2433 (58.35)	3234 (63.75)	210 (59.83)	1192 (57.14)	1897 (66.87)
Education						
High school or lower	345 (41.62)	1143 (27.41)	1088 (21.45)	127 (36.18)	503 (24.11)	695 (24.50)
Above high school	479 (57.78)	3023 (72.49)	3982 (78.49)	223 (63.53)	1583 (75.89)	2141 (75.47)
Working for money						
No	326 (39.32)	1791 (42.95)	1846 (36.39)	126 (35.90)	999 (47.89)	1198 (42.23)
Yes	503 (60.68)	2369 (56.81)	3217 (63.41)	225 (64.10)	1083 (51.92)	1639 (57.77)
Use of analgesics						
No	566 (68.28)	3021 (72.45)	3860 (76.09)	240 (68.38)	1462 (70.09)	2075 (73.14)
Yes	258 (31.12)	1149 (27.55)	1206 (23.77)	110 (31.34)	622 (29.82)	755 (26.61)
KOA symptoms						
WOMAC pain score	3.97 ± 3.91	2.67 ± 3.20	2.27 ± 3.11	4.52 ± 4.62	3.29 ± 3.66	3.25 ± 3.74
WOMAC disability score	14.10 ± 13.73	9.74 ± 10.79	8.70 ± 10.94	15.75 ± 15.14	12.19 ± 12.48	12.56 ± 12.82
WOMAC stiffness score	2.10 ± 1.84	1.64 ± 1.62	1.43 ± 1.58	2.33 ± 1.95	1.95 ± 1.78	1.92 ± 1.77
Moderate to severe symptoms						
No	388 (46.80)	2448 (58.71)	3299 (65.03)	151 (43.02)	1062 (50.91)	1432 (50.48)
Yes	441 (53.20)	1722 (41.29)	1774 (34.97)	200 (56.98)	1024 (49.09)	1405 (49.52)
KL grade						
0	601 (72.50)	2933 (70.34)	3472 (68.44)	0 (0.00)	0 (0.00)	0 (0.00)
1	228 (27.50)	1237 (29.66)	1601 (31.56)	0 (0.00)	0 (0.00)	0 (0.00)
2	0 (0.00)	0 (0.00)	0 (0.00)	252 (71.79)	1303 (62.46)	1764 (62.18)
3	0 (0.00)	0 (0.00)	0 (0.00)	99 (28.21)	783 (37.54)	1073 (37.82)
4	0 (0.00)	0 (0.00)	0 (0.00)	0 (0.00)	0 (0.00)	0 (0.00)
Study cohort						
OAI	332 (40.05)	1955 (46.88)	2610 (51.45)	223 (63.53)	1344 (64.43)	1815 (63.98)
MOST	247 (29.79)	1331 (31.92)	1931 (38.06)	105 (29.91)	633 (30.35)	958 (33.77)
CHECK	250 (30.16)	884 (21.20)	532 (10.49)	23 (6.55)	109 (5.23)	64 (2.26)

Data are presented as mean ± standard deviation or count (percentage). The percentage calculations are based on all cases, excluding missing values. *BMI* Body Mass Index, *CHECK* Cohort for Knee and Cohort for Hip, *KL* Kellgren–Lawrence, *KOA* Knee Osteoarthritis, *MOST* Multicenter Osteoarthritis Study, *OA* Osteoarthritis, *OAI* Osteoarthritis Initiative, *WOMAC* Western Ontario and McMaster Universities Arthritis Index.

**Table 2:** Baseline characteristics of knees in the cohort for investigation of the incidence of symptomatic KOA and cohort for investigation of changes in symptoms of KOA, stratified by smoking status of participants.

Baseline characteristics	Cohort for investigation of the incidence of symptomatic KOA			Cohort for investigation of changes in symptoms of KOA		
	Current smoker	Former smoker	Never smoker	Current smoker	Former smoker	Never smoker
Participants	n = 541 (7.64)	n = 2920 (41.23)	n = 3622 (51.14)	n = 139 (7.44)	n = 754 (40.34)	n = 976 (52.22)
Knees	N = 985 (7.63)	N = 5320 (41.21)	N = 6605 (51.16)	N = 196 (7.42)	N = 1054 (39.92)	N = 1390 (52.65)
Age, years	56.07 ± 7.11	61.37 ± 8.49	60.14 ± 8.66	58.34 ± 7.89	63.58 ± 8.31	62.71 ± 8.74
BMI, kg/m^2^	27.59 ± 4.77	28.36 ± 4.79	28.26 ± 4.86	30.66 ± 4.64	31.66 ± 5.81	32.16 ± 6.45
Race						
White	754 (76.55)	4678 (87.93)	5704 (86.36)	105 (53.57)	773 (73.34)	936 (67.34)
Other	231 (23.45)	642 (12.07)	901 (13.64)	91 (46.43)	281 (26.66)	454 (32.66)
Sex						
Male	431 (43.76)	2315 (43.52)	2480 (37.55)	82 (41.84)	424 (40.23)	392 (28.20)
Female	554 (56.24)	3005 (56.48)	4125 (62.45)	114 (58.16)	630 (59.77)	998 (71.80)
Education						
High school or lower	374 (37.97)	1321 (24.83)	1343 (20.33)	93 (47.45)	342 (32.45)	458 (32.95)
Above high school	606 (61.52)	3995 (75.09)	5259 (79.62)	102 (52.04)	712 (67.55)	931 (66.98)
Working for money						
No	379 (38.48)	2297 (43.18)	2456 (37.18)	74 (37.76)	548 (51.99)	607 (43.67)
Yes	606 (61.52)	3012 (56.62)	4139 (62.66)	122 (62.24)	503 (47.72)	783 (56.33)
Use of analgesics						
No	677 (68.73)	3903 (73.36)	5064 (76.67)	138 (70.41)	662 (62.81)	942 (67.77)
Yes	304 (30.86)	1417 (26.64)	1527 (23.12)	58 (29.59)	390 (37.00)	446 (32.09)
KOA symptoms						
WOMAC pain score	3.46 ± 3.79	2.26 ± 2.99	1.92 ± 2.86	7.29 ± 4.30	5.94 ± 3.52	5.86 ± 3.81
WOMAC disability score	12.16 ± 13.35	8.09 ± 10.09	7.20 ± 10.02	26.28 ± 12.31	22.75 ± 10.29	23.19 ± 10.75
WOMAC stiffness score	1.89 ± 1.80	1.45 ± 1.55	1.28 ± 1.49	3.43 ± 1.73	3.17 ± 1.61	3.09 ± 1.62
Moderate to severe symptoms						
No	549 (55.74)	3618 (68.01)	4850 (73.43)	0 (0.00)	0 (0.00)	0 (0.00)
Yes	436 (44.26)	1702 (31.99)	1755 (26.57)	196 (100.00)	1054 (100.00)	1390 (100.00)
KL grade						
0	596 (60.51)	2920 (54.89)	3457 (52.34)	0 (0.00)	0 (0.00)	0 (0.00)
1	228 (23.15)	1230 (23.12)	1597 (24.18)	0 (0.00)	0 (0.00)	0 (0.00)
2	112 (11.37)	686 (12.89)	971 (14.70)	134 (68.37)	575 (54.55)	737 (53.02)
3	39 (3.96)	376 (7.07)	461 (6.98)	51 (26.02)	359 (34.06)	529 (38.06)
4	10 (1.02)	108 (2.03)	119 (1.80)	11 (5.61)	120 (11.39)	124 (8.92)
Study cohort						
OAI	450 (45.69)	2873 (54.00)	3837 (58.09)	107 (54.59)	497 (47.15)	651 (46.83)
MOST	282 (28.63)	1544 (29.02)	2228 (33.73)	72 (36.73)	473 (44.88)	690 (49.64)
CHECK	253 (25.69)	903 (16.97)	540 (8.18)	17 (8.67)	84 (7.97)	49 (3.53)

Data are presented as mean ± standard deviation or count (percentage). The percentage calculations are based on all cases, excluding missing values. *BMI* Body Mass Index, *CHECK* Cohort for Knee and Cohort for Hip, *KL* Kellgren/Lawrence, *KOA* Knee Osteoarthritis, *MOST* Multicenter Osteoarthritis Study, *OA* Osteoarthritis, *OAI* Osteoarthritis Initiative, *WOMAC* Western Ontario and McMaster Universities Arthritis Index.

In our analyses of all six outcomes (binary and continuous), we did not adjust our estimates for body mass index (BMI) at baseline. This decision was based on the understanding that smoking may directly affect the risk of KOA as well as indirectly influence KOA through its effect on BMI (i.e., smoking affects BMI, which in turn affects KOA). By not adjusting for BMI, we aimed to capture both the direct and indirect (via BMI) effects of smoking on KOA, potentially providing a more accurate understanding of the relationship between smoking and KOA^4^. Additionally, we provided estimates from analyses adjusted for BMI at baseline to isolate the direct effect of smoking on KOA.

Mortality presents a potential source of bias in our analyses, as smoking may influence not only KOA outcomes but also mortality. To mitigate this, we explored how different assumptions about the outcomes of participants who died during follow-up might affect the associations observed. We used two distinct scenarios to assess the potential influence of mortality: a worst-case scenario and a best-case scenario. In the worst-case scenario, for the binary outcomes, we assumed that all current smokers who died during follow-up had experienced the outcomes under analysis, and for the continuous outcomes, we assumed that they had experienced the worst possible change (i.e., symptoms worsened to the greatest degree during follow-up). Those worst possible changes in symptom scores were 19 for the WOMAC subscale of pain, 51 for the WOMAC subscale of disability, and 8 for the WOMAC subscale of stiffness. In the best-case scenario, for the binary outcomes, we assumed that none of the current smokers at baseline who died during the follow-up period had experienced the outcome, and for the continuous outcomes, we assumed that they had experienced the best possible changes (i.e., symptoms got better to the greatest degree during follow-up). Those best possible changes in symptom scores were − 19 for the WOMAC subscale of pain, − 68 for the WOMAC subscale of disability, and − 8 for the WOMAC subscale of stiffness. In both scenarios (worst-case and best-case), we also addressed missing outcome data at follow-up assessments (ranging from 4.06 to 21.08% across the four cohorts, excluding deaths), as well as missing covariates related to education, employment, and use of analgesics at baseline (each comprising less than 0.2% in the four cohorts). Specifically, in the worst-case (and best-case) scenarios, we first assumed that all current smokers who died during follow-up had experienced the worst possible (and best possible) outcome and then imputed the remaining missing data. We employed a multiple imputation approach using chained equations to generate 10 separate imputed datasets for each of the worst-case and best-case scenarios. Logistic regression was used for imputing binary variables, while linear regression was used for imputing continuous variables. The imputation model included key variables such as smoking status, age, race, sex, KL grade at baseline, and study cohort to ensure that the imputation process was informed by all relevant covariates. After imputing the missing data, each of the 10 imputed datasets per scenario was analyzed separately. The results from these separate analyses were then combined using Rubin’s rules, which appropriately account for both within- and between-imputation variability. This method provides valid statistical inferences that reflect the uncertainty introduced by the imputation process, rather than merely averaging the imputed datasets.

In order to evaluate potential heterogeneity in the effect of smoking across the different study cohorts (i.e., OAI, MOST, and CHECK), we introduced interaction terms between smoking status and study cohort in our models and re-performed the analyses.

To assess the overall quality and risk of bias for each study cohort included in our meta-analysis, we employed the Newcastle–Ottawa Scale^54^ for cohort studies, which evaluates participant selection, cohort comparability, and outcome assessment (see Supplementary File).

All statistical analyses were conducted using STATA software version 18.0 (BE—Basic Edition). We set our threshold for significance to a two-tailed *P* value of less than 0.05.

## Results

### Baseline characteristics

Participants across all four cohorts were predominantly White and female (Tables 1 and 2). At baseline, compared to former or never smokers, current smokers in all four cohorts were younger, had lower BMI, were more likely to be non-White, were more frequently in the category of education of high school or less, and had higher WOMAC scores in all of the three subscales (i.e., pain, disability, and stiffness) (Tables 1 and 2).

### Association of smoking with the incidence of radiographic KOA

There was no difference in the incidence of radiographic KOA between smoking groups (Table 3). When we adjusted the analysis for baseline BMI, our findings remained consistent (Table 3). When we analyzed without adjusting for baseline KL grade, the results also remained the same or similar (results not shown). Taken together, these results indicate that the adjustments made in our analyses did not obscure any true associations.

**Table 3:** Association of smoking with the outcomes of the incidence and progression of radiographic KOA at 4-to-5 years.

	Current smoker	Former smoker	Never smoker
Cohort for investigation of the incidence of radiographic KOA	N = 829 knees	N = 4170 knees	N = 5073 knees
Outcome: Incidence of radiographic knee osteoarthritis			
Year 4-to-5			
N (%)	93 (11.22)	553 (13.26)	624 (12.30)
Effect size (95% CI)	0.91 (0.69 to 1.20)	1.08 (0.94 to 1.25)	Reference
Effect size (95% CI)*	0.97 (0.73 to 1.28)	1.08 (0.73 to 1.28)	Reference
Cohort for investigation of the progression of radiographic KOA	N = 351 knees	N = 2,086 knees	N = 2,837 knees
Outcome: Progression of radiographic knee osteoarthritis			
Year 4-to-5			
N (%)	53 (15.10)	353 (16.92)	516 (18.19)
Effect size (95% CI)	0.91 (0.64 to 1.30)	0.96 (0.81 to 1.14)	Reference
Effect size (95% CI)*	0.95 (0.67 to 1.36)	0.95 (0.80 to 1.13)	Reference

Effect sizes are odds ratio. The estimates were adjusted for sex, race, study cohort, and baseline values of age, education status, employment status, and KL grade. *Additionally adjusted for Body Mass Index (BMI) at baseline. *CI* Confidence Interval, *KL* Kellgren–Lawrence, *KOA* Knee Osteoarthritis, *OR* Odds Ratio, *SD* Standard Deviation, *WOMAC* Western Ontario and McMaster Universities Arthritis Index.

In our primary analyses, we excluded knees that had been replaced before baseline or during follow-up. Since knee replacement can indicate KOA progression, we conducted an additional analysis assuming that the knees that had been replaced between baseline and follow-up exhibited incidence of radiographic KOA. This additional analysis also found no association between smoking and the incidence of radiographic KOA (results not shown), consistent with our primary findings.

To further assess for any association between smoking status and the incidence of radiographic KOA, we examined the number of participants where neither knee, one knee, or both knees showed incidence of radiographic KOA. We found that 84.51% of participants had neither knee affected, 15.49% had one knee affected, and 0% had both knees affected. The implications of these findings will be outlined in the “Discussion” section.

When we assessed the potential impact of mortality on our results, using the worst-case scenario (where all of the current smokers who died were assumed to have had incident radiographic KOA) and the best-case scenario (where none of the current smokers who died were assumed to have had incident radiographic KOA), no differences were found between smoking groups, with one exception. In the worst-case scenario, current smokers had higher odds of the incidence of radiographic KOA compared to never smokers (OR 1.54; 95% CI 1.23–1.92), indicating a potential negative impact of smoking (Table S1).

### Association of smoking with the progression of radiographic KOA

Similar to the above findings for the incidence of radiographic KOA, there was no difference between smoking groups in the progression of radiographic KOA (Table 3). Also similar to our findings for the incidence of radiographic KOA, when we adjusted the analyses for BMI at baseline (Table 3), or without adjusting for baseline KL grade (data not shown), or when we treated knee replacement as an indicator of progression of radiographic KOA (data now shown), our findings remained the same or similar, consistent with our primary findings**.**

Similar to our analyses of the incidence of radiographic KOA, the progression of radiographic KOA did not show consistent results across both knees in participants, with only a small percentage of participants showing progression in both knees. Specifically, 90.55% of participants had neither knee progress, 8.05% had one knee progress, and only 1.40% had both knee progress. Again, the implications of these findings will be outlined in the Discussion section.

When we assessed the potential impact of mortality on our results for the progression of radiographic KOA, using the worst-case and best-case scenarios, no differences were found between smoking groups (Table S1). This finding differs from our above best-case and worst-case analyses outlined above, where current smokers had higher odds of the incidence of radiographic KOA compared to never smokers in the worst-case scenario.

### Association of smoking with the incidence of symptomatic KOA

There was no difference between smoking groups in the incidence of symptomatic KOA (Table 4). Similar to our analyses of the incidence and progression of radiographic KOA, adjusting the analysis for BMI at baseline (Table 4), or without adjusting for baseline KL grade (data not shown), or when we treated knee replacement as an indicator of incidence of symptomatic KOA (data now shown), there was also no association between smoking and the incidence of symptomatic KOA, consistent with our primary findings.

**Table 4:** Association of smoking with the outcomes of the incidence of symptomatic KOA and changes in symptoms of KOA.

	Current smoker	Former smoker	Never smoker
Cohort for investigation of the incidence of symptomatic KOA	N = 985 knees	N = 5320 knees	N = 6605 knees
Outcome: Incidence of symptomatic knee osteoarthritis			
Year 4-to-5			
N (%)	60 (6.09%)	323 (6.07)	411 (6.22)
Effect size (95% CI)	1.32 (0.94 to 1.86)	1.06 (0.88 to 1.27)	Reference
Effect size (95% CI)*	1.37 (0.98 to 1.94)	1.04 (0.87 to 1.25)	Reference
Cohort for investigation of changes in symptoms of KOA	N = 196 knees	N = 1054 knees	N = 1390 knees
Outcome: Changes in WOMAC pain score			
Year 2-to-2.5			
Unadjusted mean change from baseline ± SD	− 1.18 ± 4.48	− 0.95 ± 3.72	− 0.84 ± 3.72
Effect size (95% CI)	− 0.24 (− 0.70 to 0.21)	− 0.09 (− 0.33 to 0.15)	Reference
Effect size (95% CI)*	− 0.21 (− 0.66 to 0.24)	− 0.10 (− 0.33 to 0.14)	Reference
Year 5			
Unadjusted mean change from baseline ± SD	− 0.98 ± 5.12	− 1.06 ± 4.11	− 0.96 ± 4.14
Effect size (95% CI)	− 0.21 (− 0.84 to 0.42)	− 0.08 (− 0.41 to 0.24)	Reference
Effect size (95% CI)*	− 0.16 (− 0.79 to 0.47)	− 0.08 (− 0.40 to 0.24)	Reference
Year 7-to-8			
Unadjusted mean change from baseline ± SD	− 1.09 ± 5.28	− 0.86 ± 4.30	− 1.03 ± 4.32
Effect size (95% CI)	− 0.07 (− 0.88 to 0.75)	0.06 (− 0.36 to 0.48)	Reference
Effect size (95% CI)*	− 0.03 (− 0.85 to 0.78)	0.04 (− 0.38 to 0.46)	Reference
Outcome: Changes in WOMAC disability score			
Year 2-to-2.5			
Unadjusted mean change from baseline ± SD	− 3.83 ± 12.20	− 3.75 ± 10.97	− 3.49 ± 10.61
Effect size (95% CI)	− 0.12 (− 1.46 to 1.21)	− 0.13 (− 0.83 to 0.57)	Reference
Effect size (95% CI)*	− 0.08 (− 1.41 to 1.25)	− 0.11 (− 0.81 to 0.58)	Reference
Year 5			
Unadjusted mean change from baseline ± SD	− 3.62 ± 14.63	− 4.11 ± 12.31	− 4.58 ± 12.09
Effect size (95% CI)	0.33 (− 1.55 to 2.22)	0.63 (− 0.34 to 1.61)	Reference
Effect size (95% CI)*	0.42 (− 1.47 to 2.30)	0.67 (− 0.31 to 1.64)	Reference
Year 7-to-8			
Unadjusted mean change from baseline ± SD	− 5.56 ± 16.06	− 3.67 ± 12.26	− 4.21 ± 11.85
Effect size (95% CI)	− 1.74 (− 4.24 to 0.75)	0.61 (− 0.70 to 1.91)	Reference
Effect size (95% CI)*	− 1.69 (− 4.19 to 0.81)	0.59 (− 0.72 to 1.89)	Reference
Outcome: Changes in WOMAC stiffness score			
Year 2-to-2.5			
Unadjusted mean change from baseline ± SD	− 0.49 ± 2.16	− 0.51 ± 1.87	− 0.41 ± 1.94
Effect size (95% CI)	− 0.05 (− 0.27 to 0.18)	− 0.09 (− 0.20 to 0.03)	Reference
Effect size (95% CI)*	− 0.04 (− 0.26 to 0.19)	− 0.09 (− 0.21 to 0.03)	Reference
Year 5			
Unadjusted mean change from baseline ± SD	− 0.48 ± 2.23	− 0.56 ± 1.92	− 0.56 ± 1.94
Effect size (95% CI)	0.02 (− 0.29 to 0.33)	0.02 (− 0.14 to 0.18)	Reference
Effect size (95% CI)*	0.03 (− 0.28 to 0.34)	0.03 (− 0.14 to 0.19)	Reference
Year 7-to-8			
Unadjusted mean change from baseline ± SD	− 0.75 ± 2.37	− 0.46 ± 2.03	− 0.45 ± 2.00
Effect size (95% CI)	− 0.35 (− 0.75 to 0.58)	0.09 (− 0.12 to 0.30)	Reference
Effect size (95% CI)*	− 0.33 (− 0.74 to 0.07)	0.08 (− 0.13 to 0.29)	Reference

Effect sizes are β coefficients, except for the incidence of symptomatic KOA where effect sizes are odds ratio. The estimates were adjusted for sex, race, study cohort, and baseline values of age, education status, employment status, and use of analgesics. For the incidence of symptomatic KOA, the estimates were additionally adjusted for the baseline values of KL grade and the sum of WOMAC pain and disability score. *Additionally adjusted for Body Mass Index (BMI) at baseline. *CI* Confidence Interval, *KL* Kellgren–Lawrence, *KOA* Knee Osteoarthritis, *OR* Odds Ratio, *SD* Standard Deviation, *WOMAC* Western Ontario and McMaster Universities Arthritis Index.

Similar to our findings for the incidence and progression of radiographic KOA, the incidence of symptomatic KOA was not consistent across both knees, with 90.95% of participants having neither knee affected, 6.89% having one knee affected, and only 2.16% having both knees affected, and the implications of this observation will be outlined in the Discussion section.

As in the progression of radiographic KOA, when we assessed the potential impact of mortality on our results for the incidence of symptomatic KOA using the worst-case and best-case scenarios, no differences were found between smoking groups, with one exception. In the worst-case scenario, similar to our findings for the incidence of radiographic KOA but not for the progression of radiographic KOA as outlined above, current smokers had higher odds of incidence of symptomatic KOA compared to never smokers (OR 2.34; 95% CI 1.77–3.08), indicating a potential negative impact of smoking (Table S1).

### Association of smoking with changes in scores for the WOMAC subscales of pain, disability, and stiffness

For our analyses of the continuous variables of symptoms of KOA (pain, disability, and stiffness), we assessed the outcomes at three distinct follow-up intervals: 2-to-2.5 years; 5 years; and 7-to-8 years. These timeframes were selected because symptom fluctuations, including improvements or deteriorations, can occur over time. This contrasts with the three binary outcomes in this study (i.e., the incidence of radiographic KOA, the progression of radiographic KOA, and the incidence of symptomatic KOA), which were assessed only at one follow-up interval (i.e., the 4-to-5-year mark), as radiographic changes tend to reflect a unidirectional progression, typically worsening over time without improvement, and this 4-to-5-year timepoint was the longest follow-up with radiographic data available from all of the three study cohorts underpinning this investigation (OAI, MOST, and CHECK).

The differences between smoking groups in scores for the WOMAC subscales of pain, disability, and stiffness were not statistically significant at the 2-to-2.5-year, 5-year, and 7-to-8-year follow-up periods (Table 4). Similar to our findings for our three binary outcomes of the incidence of radiographic KOA, the progression of radiographic KOA, and the incidence of symptomatic KOA, adjusting for BMI at baseline did not have any appreciable impact on the effect sizes, which remained the same or similar (Table 4).

When we performed analyses by additionally adjusting for WOMAC scores at baseline, the results were similar to those obtained from our primary analyses, showing no statistically important difference between smoking groups (Table S2).

When assessing the impact of mortality on symptom changes, we observed contrasting effects for current smokers compared to never smokers. In the worst-case scenario, current smokers experienced worsening symptoms (increased WOMAC scores), while the best-case scenario showed symptom improvement (decreased WOMAC scores). Former smokers showed no significant differences from never smokers in either scenario (Table S1). These findings suggest smoking may have a variable effect on symptoms depending on mortality assumptions, but the effect sizes for current smokers were generally not clinically significant. The only exception was a clinically significant worsening in WOMAC stiffness in the worst-case scenario, limited to the 5-year follow-up.

### Heterogeneity, data quality, and risk of bias assessments

Our heterogeneity analyses, which incorporated interaction terms between smoking status and study cohort (i.e., OAI, MOST, or CHECK), revealed no statistically significant interactions, suggesting that the association of smoking on KOA outcomes was consistent across all three study cohorts. The quality and risk of bias assessment, conducted using the Newcastle–Ottawa Scale for cohort studies, indicated that the OAI, MOST, and CHECK cohort studies were conducted to high methodological standards (Supplementary File).

## Discussion

This IPD meta-analysis demonstrated that smoking is not linked to any beneficial outcomes for KOA as determined by radiography (with or without symptoms) over 4–5 years or for the symptoms of KOA (pain, disability, or stiffness) over 2–2.5 years, 5 years, and 7–8 years. Given the lack of evidence of any protective effect of smoking on KOA, our findings help resolve any uncertainty about the potential benefits of smoking for KOA that was suggested by previous meta-analyses that were conducted using aggregate data.

Although we found no association between smoking and beneficial KOA outcomes, we observed weak evidence of a detrimental association between smoking and the incidence of radiographic KOA and the incidence of symptomatic KOA over 4–5 years under certain analytical conditions. Specifically, when we analyzed the impact of mortality in our results, compared to never smokers, current smokers had increased odds of the incidence of radiographic KOA and of the incidence of symptomatic KOA over 4–5 years in the worst-case scenario, where we assumed that all current smokers at baseline who died during follow-up had experienced these outcomes. While this evidence may not be sufficient to make a generalized claim that smoking is associated with harmful outcomes for KOA, it does reinforce our conclusion that smoking is not associated with beneficial outcomes for KOA. Additionally, the sensitivity analysis for symptom changes showed contrasting effects for current smokers depending on the scenario analyzed. While the worst-case scenario indicated symptom worsening, the best-case scenario suggested improvement, though the effect sizes were generally small and clinically insignificant. Furthermore, these potential benefits were not accompanied by improvements in structural outcomes, as no association was found between smoking and radiographic progression. Overall, these findings suggest that smoking does not provide a consistent or clinically meaningful benefit for KOA, whether in terms of symptoms or structural outcomes.

Our conclusion that smoking is not associated with beneficial outcomes for KOA is further corroborated by our comparison of knees within participants. We observed that when a particular outcome was observed (e.g., the incidence of radiographic KOA, etc.), it was overwhelmingly more commonly observed in only one knee per participant rather than in both knees of the participant, providing further evidence that smoking does not have a significant impact on KOA. Specifically, while 15.49%, 8.05%, and 6.89% of participants had only one knee exhibiting the incidence of radiographic KOA, the progression of radiographic KOA, or the incidence of symptomatic KOA over the 4-to-5-year follow-up, respectively, only 0%, 1.40%, and 2.16% of participants had both knees exhibiting these outcomes. If smoking had a substantial effect—whether protective or harmful—we would expect a more consistent pattern of observation of outcomes in both knees. This finding is in keeping with the premise that smoking is not associated with any benefit for the incidence or progression of KOA.

Our findings diverge from the previously published meta-analyses on this topic^31–34^, all four of which reported a protective association between smoking and the development of KOA^31–33^ and between smoking and the progression of KOA^34^. Nonetheless, these earlier studies may have been subject to inherent biases or limitations. For example, publication bias may have influenced the findings of the first meta-analysis^31^, while combining current and former smokers may have led to inaccurate estimates of the association between smoking and KOA in the second meta-analysis^32^. The third meta-analysis^33^ relied on data from both case–control and cohort studies. However, the inherent selection bias in case–control studies might have affected the accuracy of these estimates. When the analysis was restricted to cohort studies in that third study^33^, no significant association was observed between smoking and KOA. This was corroborated by the authors of the third study^33^ in a subsequent meta-analysis^55^, which only included cohort studies, not case–control studies. In that subsequent meta-analysis^55^, smoking was not associated with KOA. The last of the four previously published meta-analyses^34^, which investigated the progression of KOA, included five studies and showed an association between smoking and beneficial outcomes for radiographic KOA. The five studies included in that meta-analysis^34^ differed in their definitions of radiographic KOA progression (e.g., any increase in KL grade, progression to a KL grade of ≥ 2, or progression to a KL grade of ≥ 3). These variations in defining progression may have contributed to the observed association between smoking and beneficial outcomes for KOA.

The strength of our study lies in the use of IPD, allowing for more accurate and dependable estimates than those found in previous meta-analyses^31–34^. Additionally, our risk of bias assessment using the Newcastle–Ottawa Scale found that the cohort studies included in the present IPD meta-analysis had high methodological quality in terms of participant selection, comparability of cohorts, and outcome assessment**.** However, we recognize certain limitations to our study. Primarily, our findings are associative due to the observational nature of the underlying cohort studies included in our IPD meta-analyses. Additionally, the current study was not registered upfront in a registry such as PROSPERO, which could have provided a predefined framework and potentially minimized the risk of bias. However, several measures were implemented to reduce bias in the current study. These included conducting sensitivity analyses to assess the robustness of findings under different assumptions, using multiple imputations to handle missing data, performing adjustments for key baseline variables to account for potential confounders, and including the PRISMA-IPD checklist as a supplementary file to enhance the transparency of the study. Secondly, although we adjusted our results for underlying study cohorts (i.e., OAI, MOST, and CHECK), there may still exist uncontrolled differences in factors such as patient recruitment, study setting, countries, knee malalignment, treatment information such as physiotherapy during follow-up, and any other confounding factors between smokers and non-smokers. Importantly, we conducted additional analyses to assess whether the association between smoking and KOA outcomes varied across the different study cohorts by including interaction terms between smoking status and study cohort. The results showed no statistically significant interactions, suggesting that the effect of smoking is consistent across these study cohorts, thereby supporting the generalizability of our findings. Thirdly, the smoking status of participants was determined from self-reported data. While self-reported smoking status in adults is considered reliable^56^, potential misclassification or reporting bias may have led to underestimation or overestimation of the associations observed in our study. As a fourth limitation, the study cohorts underpinning our IPD meta-analyses were predominantly made up of White participants aged 45 years or older, which may limit the broader applicability of our findings. Future prospective longitudinal studies with more diverse populations, including individuals from different ethnic backgrounds and younger age groups, may help to determine whether the lack of association between smoking and KOA outcomes holds across different demographics. As a fifth limitation, participants in the CHECK study were not specifically asked to indicate which joint (knee/hip; left/right) their WOMAC assessments referred to. In MOST, WOMAC disability scores, but not WOMAC pain and stiffness scores, reflected the highest level of disability reported across both knees, rather than assessing each knee individually. While the impact of these limitations on the overall analysis is uncertain, we applied conservative assumptions by considering the highest reported disability scores in MOST and treating unspecified joints in CHECK as referring to the most affected joint. This approach means that our estimates remain cautious and unlikely to overstate any associations. An additional limitation is that our analyses did not account for the duration and quantity of smoking throughout participant’s lifetime, as this data was not readily available in all of the three study cohorts used for our IPD meta-analyses. Furthermore, while our IPD meta-analyses offer the advantages of detailed and standardized data analysis, it is important to acknowledge that aggregate data meta-analyses can incorporate a broader range of studies. This limitation arises because IPD meta-analyses require studies to be similar enough in design and data collection to allow for the combination and standardization of individual-level data.

In conclusion, our study, leveraging three large cohorts and the advantages of IPD meta-analysis, finds no evidence that smoking offers a protective effect against KOA, refuting the notion that smoking may benefit joint health.

## Supplementary Information

Supplementary Information.

## Data Availability

The datasets were derived from sources in the public domain: OAI public use data sets are available through the National Institute of Mental Health Data Archive, MOST public use data sets are available through the NIA Aging Research Biobank, and CHECK public use data sets are available through the https://easy.dans.knaw.nl/ui/home.
