# Supplementary material for: Association of smoking with knee osteoarthritis structural defects and symptoms: an individual participant data meta-analysis
Source: Sci Rep. 2024 Nov 22;14:29021. doi: 10.1038/s41598-024-80345-x (PMC11584879; doi:10.1038/s41598-024-80345-x)
Supplement: Supplementary file 1 — Supplementary Information. [file 41598_2024_80345_MOESM1_ESM.docx]

**Association of Smoking with Knee Osteoarthritis Structural Defects and Symptoms: An Individual Participant Data Meta-Analysis**

Zubeyir Salis*^1^, Amanda Sainsbury^2^

^1^. Division of Rheumatology, Geneva University Hospital and Faculty of Medicine, University of Geneva, Geneva, Switzerland

^2^. The University of Western Australia, School of Human Sciences, Perth, WA, Australia

***Corresponding author: Zubeyir Salis**

Email: Zubeyir.Salis@etu.unige.ch

Address: HUG Av. de Beau-Séjour 26, 1206 Genève Suisse

Table of contents

[**NEWCASTLE - OTTAWA Quality Assessment Scale Cohort Studies** 2](#_Toc180144442)

[**Table S1.** Sensitivity analysis of impact of mortality in the estimates. 6](#_Toc180144443)

[**Table S2.** Sensitivity analysis by additionally adjusting for baseline symptom scores. 10](#_Toc180144444)

# **NEWCASTLE - OTTAWA Quality Assessment Scale Cohort Studies**

A) Symptomatic Outcomes

**Osteoarthritis Initiative (OAI)**

**Selection**

1. **Representativeness of the exposed cohort**
   a) truly representative of the average population at risk of KOA in the community. ★
2. **Selection of the non-exposed cohort**
   a) drawn from the same community as the exposed cohort. ★
3. **Ascertainment of exposure**
   c) written self-report (as smoking status is typically self-reported in the OAI).
4. **Demonstration that outcome of interest was not present at start of study**
   a) yes. ★

**Comparability**

1. **Comparability of cohorts on the basis of the design or analysis**
   a) study controls for baseline factors like age, sex, race. ★
   b) study controls for additional factors like education and employment status. ★

**Outcome (Symptomatic outcomes)**

1. **Assessment of outcome**
   c) self-report (for symptomatic KOA, outcome assessment is often through self-report).
2. **Was follow-up long enough for outcomes to occur?**
   a) yes (follow-up was long enough to observe the incidence/progression of KOA). ★
3. **Adequacy of follow-up of cohorts**
   a) complete follow-up – all subjects accounted for (or nearly complete, with minimal loss to follow-up). ★

**Multicenter Osteoarthritis Study (MOST)**

**Selection**

1. **Representativeness of the exposed cohort**
   a) truly representative of the average population at risk of KOA in the community. ★
2. **Selection of the non-exposed cohort**
   a) drawn from the same community as the exposed cohort. ★
3. **Ascertainment of exposure**
   c) written self-report (similar to OAI, smoking status is typically self-reported in MOST).
4. **Demonstration that outcome of interest was not present at start of study**
   a) yes. ★

**Comparability**

1. **Comparability of cohorts on the basis of the design or analysis**
   a) study controls for baseline factors like age, sex, race. ★
   b) study controls for additional factors like education and employment status. ★

**Outcome (Symptomatic outcomes)**

1. **Assessment of outcome**
   c) self-report (WOMAC scores are self-reported).
2. **Was follow-up long enough for outcomes to occur?**
   a) yes (follow-up was adequate for observing KOA outcomes). ★
3. **Adequacy of follow-up of cohorts**
   a) complete follow-up – all subjects accounted for (or nearly complete, with minimal loss to follow-up). ★

**Cohort Hip and Cohort Knee (CHECK)**

**Selection**

1. **Representativeness of the exposed cohort**
   b) somewhat representative of the average population at risk of KOA in the community (CHECK participants are selected based on early symptoms, which may not represent the general population at risk). ★
2. **Selection of the non-exposed cohort**
   a) drawn from the same community as the exposed cohort. ★
3. **Ascertainment of exposure**
   c) written self-report (similar to OAI and MOST, smoking status is self-reported in CHECK).
4. **Demonstration that outcome of interest was not present at start of study**
   a) yes. ★

**Comparability**

1. **Comparability of cohorts on the basis of the design or analysis**
   a) study controls for baseline factors like age, sex, race. ★
   b) study controls for additional factors like education and employment status. ★

**Outcome (Symptomatic outcomes)**

1. **Assessment of outcome**
   c) self-report (WOMAC scores are self-reported).
2. **Was follow-up long enough for outcomes to occur?**
   a) yes (adequate follow-up for observing KOA outcomes). ★
3. **Adequacy of follow-up of cohorts**
   b) subjects lost to follow-up unlikely to introduce bias – small number lost (a slightly higher attrition rate might be present in CHECK compared to OAI and MOST, but it's unlikely to bias results significantly). ★

**B) Radiographic Outcomes**

**Osteoarthritis Initiative (OAI)**

**Selection**

1. **Representativeness of the exposed cohort**
   a) truly representative of the average population at risk of KOA in the community. ★
2. **Selection of the non-exposed cohort**
   a) drawn from the same community as the exposed cohort. ★
3. **Ascertainment of exposure**
   c) written self-report (as smoking status is typically self-reported in the OAI).
4. **Demonstration that outcome of interest was not present at start of study**
   a) yes. ★

**Comparability**

1. **Comparability of cohorts on the basis of the design or analysis**
   a) study controls for baseline factors like age, sex, race. ★
   b) study controls for additional factors like education and employment status. ★

**Outcome**

1. **Assessment of outcome**
   a) independent blind assessment ★.
2. **Was follow-up long enough for outcomes to occur?**
   a) yes (follow-up was long enough to observe the incidence/progression of KOA). ★
3. **Adequacy of follow-up of cohorts**
   a) complete follow-up – all subjects accounted for (or nearly complete, with minimal loss to follow-up). ★

**Multicenter Osteoarthritis Study (MOST)**

**Selection**

1. **Representativeness of the exposed cohort**
   a) truly representative of the average population at risk of KOA in the community. ★
2. **Selection of the non-exposed cohort**
   a) drawn from the same community as the exposed cohort. ★
3. **Ascertainment of exposure**
   c) written self-report (similar to OAI, smoking status is typically self-reported in MOST).
4. **Demonstration that outcome of interest was not present at start of study**
   a) yes. ★

**Comparability**

1. **Comparability of cohorts on the basis of the design or analysis**
   a) study controls for baseline factors like age, sex, race. ★
   b) study controls for additional factors like education and employment status. ★

**Outcome**

1. **Assessment of outcome**
   a) independent blind assessment ★
2. **Was follow-up long enough for outcomes to occur?**
   a) yes (follow-up was adequate for observing KOA outcomes). ★
3. **Adequacy of follow-up of cohorts**
   a) complete follow-up – all subjects accounted for (or nearly complete, with minimal loss to follow-up). ★

**Cohort Hip and Cohort Knee (CHECK)**

**Selection**

1. **Representativeness of the exposed cohort**
   b) somewhat representative of the average population at risk of KOA in the community (CHECK participants are selected based on early symptoms, which may not represent the general population at risk). ★
2. **Selection of the non-exposed cohort**
   a) drawn from the same community as the exposed cohort. ★
3. **Ascertainment of exposure**
   c) written self-report (similar to OAI and MOST, smoking status is self-reported in CHECK).
4. **Demonstration that outcome of interest was not present at start of study**
   a) yes. ★

**Comparability**

1. **Comparability of cohorts on the basis of the design or analysis**
   a) study controls for baseline factors like age, sex, race. ★
   b) study controls for additional factors like education and employment status. ★

**Outcome**

1. **Assessment of outcome**
   a) independent blind assessment ★
2. **Was follow-up long enough for outcomes to occur?**
   a) yes (adequate follow-up for observing KOA outcomes). ★
3. **Adequacy of follow-up of cohorts**
   b) subjects lost to follow-up unlikely to introduce bias – small number lost (a slightly higher attrition rate might be present in CHECK compared to OAI and MOST, but it's unlikely to bias results significantly). ★

**SUMMARY**

|  | Symptomatic Outcomes | | | Radiographic Outcomes | | |
| --- | --- | --- | --- | --- | --- | --- |
|  | Selection | Comparability | Outcome | Selection | Comparability | Outcome |
| OAI | ★★★ | ★★ | ★★ | ★★★ | ★★ | ★★★ |
| MOST | ★★★ | ★★ | ★★ | ★★★ | ★★ | ★★★ |
| CHECK | ★★★ | ★★ | ★★ | ★★★ | ★★ | ★★★ |

These scores indicate that OAI, MOST, and CHECK studies have similar quality ratings. Overall, all three cohorts are generally robust, with a good overall risk of bias profile.

# **Table S1.** Sensitivity analysis of the impact of mortality in the estimates.

|  | **Best case** | | | **Worst-case** | | |
| --- | --- | --- | --- | --- | --- | --- |
|  | **Current**  **smoker** | **Former**  **smoker** | **Never**  **smoker** | **Current**  **smoker** | **Former**  **smoker** | **Never**  **smoker** |
| **Cohort for investigation of the incidence of radiographic KOA** | N = 829 knees | N = 4,170 knees | N = 5,073 knees | N = 829 knees | N = 4,170 knees | N = 5,073 knees |
| **Number of knees from participants who died during the 4 to 5 years follow-up** | 77 knees  (9.29%) | 303 knees  (7.27%) | 243 knees  (4.79%) | 77 knees  (9.29%) | 303 knees  (7.27%) | 243 knees  (4.79%) |
| Outcome: Incidence of radiographic KOA | | | | | | |
| Year 4-to-5 | | | | | | |
| Effect size (95% CI) | 0.92  (0.71 to 1.19) | 1.09  (0.95 to 1.26) | Reference | **1.54**  **(1.23 to 1.92)** | 1.12  (0.98 to 1.29) | Reference |
| **Cohort for the progression of radiographic KOA** | N=351 knees | N=2,086 knees | N=2,837 knees | N=351 knees | N=2,086 knees | N=2,837 knees |
| **Number of knees from participants who died during the 4 to 5 years follow-up** | 22 knees  (6.27%) | 171 knees  (8.20%) | 145 knees  (5.11%) | 22 knees  (6.27%) | 171 knees  (8.20%) | 145 knees  (5.11%) |
| Outcome: Progression of radiographic KOA | | | | | | |
| Year 4-to-5 | | | | | | |
| Effect size (95% CI) | 0.87  (0.62 to 1.23) | 0.96  (0.80 to 1.15) | Reference | 1.07  (0.77 to 1.50) | 0.97  (0.82 to 1.13) | Reference |
| **Cohort for the investigation of incidence of symptomatic KOA** | N=985 knees | N=5,320 knees | N=6,605 knees | N=985 knees | N=5,320 knees | N=6,605 knees |
| **Number of knees from participants who died during the 4 to 5 years follow-up** | 81 knees  (8.22%) | 387 knees  (7.27%) | 329 knees  (4.98%) | 81 knees  (8.22%) | 387 knees  (7.27%) | 329 knees  (4.98%) |
| Outcome: Incidence of symptomatic KOA | | | | | | |
| Year 4-to-5 | | | | | | |
| Effect size (95% CI) | 1.28  (0.92 to 1.77) | 1.06  (0.89 to 1.26) | Reference | **2.34**  **(1.77 to 3.08)** | 1.06  (0.87 to 1.28) | Reference |
| **Cohort for the investigation of changes in symptoms of KOA** | N=196 knees | N=1,054 knees | N=1,390 knees | N=196 knees | N=1,054 knees | N=1,390 knees |
| **Number of knees from participants who died during the 7 to 8 years follow-up period** | 16 knees  (8.16%) | 109 knees  (10.34%) | 78 knees  (5.61%) | 16 knees  (8.16%) | 109 knees  (10.34%) | 78 knees  (5.61%) |
| Outcome: Changes in WOMAC pain score | | | | | | |
| Year 2-to-2.5 | | | | | | |
| Effect size  (95% CI)) | **-1.34**  **(-1.85 to -0.83)** | -0.08  (-0.34 to 0.17) | Reference | **1.01**  **(0.52 to 1.50**) | -0.05  (-0.30 to 0.21) | Reference |
| Year 5 | | | | | | |
| Effect size (95% CI) | **-0.83**  **(-1.44 to -0.22)** | -0.10  (-0.41 to 0.22) | Reference | **1.07**  **(0.42 to 1.73)** | 0.04  (-0.28 to 0.35) | Reference |
| Year 7-to-8 | | | | | | |
| Effect size (95% CI) | -**1.23**  **(-1.96 to -0.50**) | 0.09  (-0.31 to 0.49) | Reference | **1.44**  **(0.68 to 2.19)** | 0.21  (-0.20 to 0.61) | Reference |
| Outcome: Changes in WOMAC disability score | | | | | | |
| Year 2-to-2.5 | | | | | | |
| Effect size (95% CI) | **-4.25**  **(-5.70 to -2.79)** | -0.23  (-1.01 to 0.55) | Reference | **3.09**  **(1.58 to 4.60)** | -0.05  (-0.88 to 0.77) | Reference |
| Year 5 | | | | | | |
| Effect size (95% CI) | **-2.52**  **(-4.42 to -0.62)** | 0.32  (-0.70 to 1.35) | Reference | **3.53**  **(1.73 to 5.34)** | 0.61  (-0.31 to 1.53) | Reference |
| Year 7-to-8 | | | | | | |
| Effect size (95% CI) | **-5.46**  **(-7.59 to -3.34)** | 0.02  (-1.16 to 1.20) | Reference | **3.08**  **(0.91 to 5.25)** | 0.64  (-0.47 to 1.74) | Reference |
| Outcome: Changes in WOMAC stiffness score | | | | | | |
| Year 2-to-2.5 | | | | | | |
| Effect size (95% CI) | **-0.53**  **(-0.77 to -0.30)** | -0.10  (-0.23 to 0.02) | Reference | **0.46**  **(0.21 to 0.71)** | -0.07  (-0.19 to 0.06) | Reference |
| Year 5 | | | | | | |
| Effect size (95% CI) | **-0.37**  **(-0.66 to -0.08)**) | -0.01  (-0.15 to 0.14) | Reference | **0.55***  **(0.27 to 0.83**) | 0.06  (-0.10 to 0.23) | Reference |
| Year 7-to-8 | | | | | | |
| Effect size (95% CI)* | **-0.76**  **(-1.16 to -0.37**) | -0.03  (-0.22 to 0.16) | Reference | **0.49**  **(0.12 to 0.86)** | -0.03  (-0.16 to 0.21) | Reference |

Effect sizes are β coefficients, except for the incidence of radiographic KOA, the progression of radiographic KOA, and the incidence of symptomatic KOA, where effect sizes are odds ratio. The estimates were adjusted for sex, race, study cohort, and baseline values of age, education status, and employment status. For the incidence and progression of radiographic KOA the estimates were additionally adjusted for the baseline value of KL grade. For the incidence of symptomatic KOA, the estimates were additionally adjusted for the baseline values of KL grade, the sum of WOMAC pain and disability score, and the use of analgesics. For the changes in symptomatic KOA, the estimates were additionally adjusted for analgesic use.

* denotes clinically significant symptoms. The minimal clinically important difference (MCID) for symptom worsening was defined as: ≥ 1.28 units for pain, ≥ 7.00 units for disability, and ≥ 0.23 units for stiffness on the WOMAC scale [1]. A change was considered clinically significant for worsening if the lower bound of the CI exceeded the respective MCID. For symptom improvement, the MCID values were ≥ 1.66 units for pain, ≥ 5.44 units for disability, and ≥ 0.81 units for stiffness [1]. A change was considered clinically significant for improvement if the upper bound of the CI exceeded the respective MCID.

CI: Confidence Interval; KL: Kellgren-Lawrence; KOA: Knee Osteoarthritis; OR: Odds Ratio; SD: Standard Deviation; WOMAC: Western Ontario and McMaster Universities Arthritis Index.

1. Angst, F., et al., *Minimal clinically important rehabilitation effects in patients with osteoarthritis of the lower extremities.* J Rheumatol, 2002. **29**(1): p. 131-8.

# **Table S2.** Sensitivity analysis by additionally adjusting for baseline symptom scores.

|  | **Current smoker** | **Former smoker** | **Never smoker** |
| --- | --- | --- | --- |
| **Cohort for investigation of changes in symptoms of KOA** | N=196 knees | N=1,054 knees | N=1,390 knees |
| Outcome: Changes in WOMAC pain score | | | |
| Year 2-to-2.5 | | | |
| Effect size (95% CI) | −0.07 (-0.50 to 0.36) | −0.07 (-0.29 to 0.16) | Reference |
| Year 5 | | | |
| Effect size (95% CI) | 0.01< (-0.58 to 0.59) | −0.06 (-0.36 to 0.24) | Reference |
| Year 7-to-8 | | | |
| Effect size (95% CI) | 0.15 (-0.60 to 0.90) | 0.10 (-0.29 to 0.49) | Reference |
| **WOMAC disability score** | | | |
| Year 2-to-2.5 | | | |
| Effect size (95% CI) | 0.18 (-1.12 to 1.48) | −0.15 (-0.83 to 0.53) | Reference |
| Year 5 | | | |
| Effect size (95% CI) | 0.66 (-1.16 to 2.49) | 0.60 (-0.35 to 1.54) | Reference |
| Year 7-to-8 | | | |
| Effect size (95% CI) | −1.39 (-3.81 to 1.03) | 0.58 (-0.68 to 1.84) | Reference |
| **WOMAC stiffness score** |  |  |  |
| Year 2-to-2.5 | | | |
| Effect size (95% CI) | 0.02 (-0.19 to 0.22) | −0.05 (-0.16 to 0.06) | Reference |
| Year 5 | | | |
| Effect size (95% CI) | 0.09 (-0.19 to 0.38) | 0.06 (-0.09 to 0.20) | Reference |
| Year 7-to-8 | | | |
| Effect size (95% CI)* | −0.27 (-0.64 to 0.10) | 0.11 (-0.08 to 0.31) | Reference |

Effect sizes are β coefficients. The estimates were adjusted for sex, race, study cohort, and baseline values of age, education status, employment status, analgesic use, and respective baseline symptom score. CI: Confidence Interval; KOA: Knee Osteoarthritis; OR: Odds Ratio; SD: Standard Deviation; WOMAC: Western Ontario and McMaster Universities Arthritis Index.

END OF DOCUMENT
